# Impact of Face-Recognition-Based Access Control System on College Students’ Sense of School Identity and Belonging During COVID-19 Pandemic

**DOI:** 10.3389/fpsyg.2022.808189

**Published:** 2022-02-16

**Authors:** Qiang Wang, Lan Hou, Jon-Chao Hong, Xiantong Yang, Mengmeng Zhang

**Affiliations:** ^1^College of Elementary Education and College of Education, Capital Normal University, Beijing, China; ^2^Institute for Research Excellence in Learning Sciences, National Taiwan Normal University, Taipei, Taiwan; ^3^Faculty of Psychology, Beijing Normal University, Beijing, China; ^4^School of Education, Minzu University of China, Beijing, China

**Keywords:** school identity, school belonging, TAM, halo effect, COVID-19

## Abstract

In the context of coronavirus pandemic (COVID-19), the face-recognition-based access control system (FACS) has been intensively adopted to protect students’ and teachers’ health and safety in school. However, the impact of FACS, as a new technology, on students’ attitude toward accepting FACS has remained unknown from the psychological halo effect. Drawn on “halo effect” theory where psychological effects affect the sense of social identity and belonging, the present study explored college students’ sense of school identity and belonging in using FACS during COVID-19 based on the technology acceptance model (TAM). Data collected from 391 college students was analyzed using SEM to verify the relationship among perceived usefulness (PU), perceived ease of use (PEU), intention to use (IU), school identity, and school belonging. The results show that PU and PEU can positively predict IU, and consequentially can positively predict school identity and school belonging. Our study expands the application of halo effect theory to study FACS acceptance based on TAM, and provides strong evidence to support the effect of school FACS during the pandemic. The findings of this study also suggest that FACS acceptance can enhance students’ sense of school identity and belonging.

## Introduction

The face recognition system is an information system for biometric recognition by collecting facial feature information (Viola and Jones, 2004; Shen and Bai, 2006; Adjabi et al., 2020). In the past few decades, with the rapid development of information technology, products supported by face recognition technology have been widely used in various fields (Kontellis et al., 2021; Sakshi et al., 2021), such as education (Yan, 2020), medical treatment (Cantoni et al., 2018), military (Mann, 2019), biology (Iqbal et al., 2000), Internet of things (Hu et al., 2018), and so on. The face-recognition-based access control system (FACS) can protect the user’s privacy in the process of identity recognition (Chamikara et al., 2020). The outbreak of the coronavirus pandemic in 2019 (COVID-19) has threatened people’s lives and safety, and many countries have begun to adopt protective measures based on information system to protect people’s health and safety (Schmidt et al., 2021). To better protect the health and safety of students, some colleges and universities in China have also set up FACS to prevent irrelevant personnel from entering the school. This has effectively curbed the spread of the epidemic and strengthened the protection of teachers and students in the school (Wang et al., 2020). However, the psychological effect on college students in using FACS has not been extendedly studied. Therefore, based on the psychological effect of halo effect, this study explored students’ school identity under the framework of technology acceptance model (TAM) in using FACS.

Although FACS protects students at school by setting up isolation barriers, will this measure affect the students’ concept of the school? The halo effect means the impression of one area could impact the opinion/perception of another area (Boatwright et al., 2008). A person’s overall cognition or judgment of somethings often affect the overall impression of things (Thorndike, 1920). Based on the halo effect, it can be further inferred that the acceptance of FACS, as one part of the school, may affect the students’ view of the school. Lachman and Bass (1985) found that the evaluation of an individual’s specific characteristics related to a person affects the overall impression of that person. Similar studies have also found that students’ perception of the school atmosphere will have an impact on the school’s overall identity (Ullman, 2015). However, there is still a lack of research on the impact of FACS on students’ sense of belonging in school, therefore, there is an emphasizing on FACS on students’ sense of belonging in this study.

School belonging is the degree to which students feel personally accepted, respected, included, and supported by group members in the school social group (Goodnow, 1992; Goodenow, 1993). Studies have shown that the level of a student’s sense of school belongings has a great impact on students’ learning motivation and academic achievement (Barbieri and Miller-Cotto, 2021). Students’ sense of school belonging was positively linked to character strength of kindness (Lee and Huang, 2021). On the contrary, students who lack a sense of belonging to the school show a hostile and alienated attitude toward the school, with overwhelming loneliness, poor academic performance, higher crime and dropout rates (Gottfredson et al., 1993; Voelkl, 1997). The lack of sense of belonging in school serves as a barrier to developing positive relationships with supportive approaches at school (Johnson et al., 2020). Taken FACS as a supportive approach to facilitate school administration, how students’ sense of belonging related to the acceptance of FACS is explored in this study. Therefore, in order to examine the impact of college students’ use of FACS on their sense of belonging to the school, this study explores the relationship between the system’s perceived ease of use (PEU), perceived usefulness (PU), intention to use (IU), and school identity, school belonging on the basis of the TAM framework, the halo effect and social information processing theory, and provides evidence support for the application and influence of the access control system in the field of educational practice. Overall, in this study, we expected to expands the application scenarios and follow-up conditions of TAM, and promote the use of school access control systems during the pandemic.

### Relationship Between Technology Acceptance Model and School Identity

TAM is used to measure personal acceptance and adoption of technology (Venkatesh et al., 2003). It is widely used and tested to study the impact and effects of technology uses in the information science (Davis et al., 1989; Saadé and Kira, 2007; Teo, 2009). With its robust, powerful and concise characteristics, TAM has been strongly appreciated for predicting user acceptance of technology (Venkatesh and Davis, 2000).

In TAM, PU refers to the degree to which an individual believes that the use of technology can increase his/her productivity (Davis et al., 1989; Teo and Noyes, 2011). That is, people perceived that these applications can help them do their work better. The advantages of the information technology system will be offset by applications that are not easy to operate (Davis, 1989), which is also known as the PEU. On the one hand, factors such as the availability of information on the website and the ease of understanding information will predict people’s PEU of the website (Lederer et al., 2000). That is, PEU will predict PU. On the other hand, a person’s PU and PEU may also predict their IU (Davis, 1989). For example, farmers’ PU of intelligent agricultural technology predicts their willingness to adopt the technology (Jauk et al., 2021). The tendency of healthcare professionals to use visual inspection applications depends on the application’s characteristics such as visualization and easy-to-understand information presented (Jauk et al., 2021).

Within TAM, different dimensions are associative with each other. First, PU is predicted by PEU. The research results of college students’ actual use of mobile learning management systems show that PEU could positively predict PU (Joo et al., 2016). In addition, PU and PEU also predict IU. Research has found that PU is closely related to IU and plays a decisive role in the use of technology and systems (Davis et al., 1989). Moreover, learners’ PU and PEU positively *affected* the willingness to use mobile learning through learning satisfaction (Joo et al., 2014). Therefore, we propose three theoretical hypotheses within TAM:

H1: PEU positively affects PU;

H2: PU positively affects IU;

H3: PEU positively affects IU;

TAM also expands outward and affects students’ school identity. According to the definition of social identity in social psychology, school identity is described as group identity, which is the identity of college students’ belonging, emotional sustenance and values, and it is also the embodiment of identity in the school field (Schwartz, 2001). From a cognitive point of view, school identity is a college student’s personal cognition of the identity of being a member of the university, which is consistent with the school’s values. From an emotional point of view, school identity is the loyalty and pride formed in accord with a student’s expectations of the university. From the behavioral perspective, school identity is a process of forming corresponding words and deeds based on the recognition of university values. Therefore, school identity can be divided into four dimensions (Ding, 2012): *group cognition* which refers to the recognition of group characteristics (Tajfel, 1969; Jackson and Smith, 1999); e*motional dimension* which is defined as the emotional attachment to the school group and school organization (Phinney, 1990); *evaluation dimension* which is defined as college students’ evaluation of the quality of their school (Ellemers et al., 1999); and *autonomous behavior* refers to the behavior of independently maintaining the reputation of the school (Jackson, 2002). In this study, we measure college students’ school identity from these four aspects.

Students’ school identity is built on their understanding of the basic elements of the school. Among them, a study highlighted the role of the educational context in identity formation processes (Kaplan and Flum, 2012), which pointed that the school environment (e.g., school safety) forms students’ concept of who they are and who they want to be. For example, the school social climate (e.g., school entrance guard) promoted high-school students’ identity development.

From the sociocultural perspective, schools provide students with an important context for the development of students’ school identity and school teachers can help students increase their school identity (Schachter and Rich, 2011; Kaplan and Flum, 2012; Rich and Schachter, 2012). Verhoeven et al. (2019) suggested that teachers’ teaching strategies, teachers’ expectations, peer norms, and classroom atmosphere will affect adolescents’ school identity (Schachter and Rich, 2011). In turn, this context-related identity (such as students, scientists) has a persistent prediction of performance and achievement (Osborne and Walker, 2006). However, with the application of FACS in colleges and universities, there is still a certain research gap on the impact of this measure on students’ school identity.

According to the halo effect, people’s perception of a part of a thing will affect their judgment of the same thing. Specifically, ratings are clearly affected by a distinct tendency to think that the person is generally very good or very bad, and to influence judgments of quality through this general feeling (Lachman and Bass, 1985). Since FACS changes the local environment of the school, this partial change may affect students’ perception of the overall school (Thorndike, 1920). What’s more, students’ perception of the school’s local atmosphere will affect their perception of the general school environment (Ullman, 2015). Additionally, the social information processing theory believes that an individual will collect information from his social environment (such as the service function of school facilities) and form an individual’s general perception of school policies, values, and norms on the basis of the information (Salancik and Pfeffer, 1978). Students’ perceptions of their school climate (e.g., sense of security) have the effect on their connection to their school community which is related to school identity (Ullman, 2015). It could be inferred that individual perception of school safety would be related to acceptance of the FACS because it serves as an important part in school climate. Accordingly, students’ technical acceptance of the school’s access control system service facilities (such as PU, PEU, and IU) may lead to the formation of students’ comprehensive judgments of the school’s environment and school atmosphere, which in turn affects students’ identity of the environment and atmosphere in school. In view of those above reasons, we propose hypotheses 4∼6:

H4: PU positively affects school identity;

H5: PEU positively affects school identity;

H6: IU positively affects school identity.

### Relationship Between School Identity and School Belonging

School belonging refers to the personal connection with school or school members, and the feeling of integration with other people in the school (Pittman and Richmond, 2007). School belonging includes four dimensions (Mueller, 2008): *sense of belonging with peers*, *instructor support*, *engagement in the community*, and *relatedness of self with school*. As it is shown in the study, school belonging plays a very important role in the mental and physical health of college students (Barden et al., 1985). College students build school belonging by interacting with their peers in the broader cultural climate every day, which is not only closely related to the students’ school identity, but also has a positive effect on their academic success (Offidani-Bertrand et al., 2019) and life satisfaction (Arslan et al., 2020), so studying school belonging is of great significance to help them establish a high-quality community life.

Studies have found that there is a close connection between identity and belonging. Social identity refers to an individual’s knowledge about his belonging to a certain social group and the emotional/value significance brought by the role as a group member (Tajfel, 1972, 1974). According to the theory of social identity, if people are dissatisfied with their current social identity in the group, people will try to leave the group and join a more favorable one (Brown et al., 1980; Tajfel, 1982), and higher sense of identity also indicates stronger sense of belonging. The latest research has found that minority students often make false internal attributions—they are not good at learning science—because of their ethnic identity and thus reduce participation in science classes (Chen et al., 2021). In contrast, a strong sense of scientific identity can be a psychological resource to enhance students’ sense of belonging in science courses, as well as to promote the academic performance of minority students. Legette and Kurtz-Costes (2021) studied the changes in the sense of belonging between excellent students and ordinary students in 6th Grade in math, confirming the predictive effect of academic identity which could been seen a part of school identity on the changes of the sense of belonging in the school. In addition, Koenka et al. provided further evidence for the relationship between school identity and school belonging, that is, ethnic identity certainly predicted a stronger sense of school belonging (Koenka et al., 2020). Although school identity is closely related to school belonging, more direct evidence is needed. Based on the results from these previous studies, we propose hypothesis 7:

H7: school identity positively predicts school belonging.

### Present Study

Given that school identity and school belonging are critical to students’ academic adaptation, life satisfaction and personal happiness, and that FACS has quietly changed the environmental ecology of colleges and universities during the COVID-19 pandemic, this change may have a significant impact on the above two important school perceptions (school identity and school belonging). Hence this research attempts to explore the relationships between TAM, school identity and school belonging based upon the halo effect.

Comprehensive analysis of previous studies has found that the internal factors and antecedents of the TAM often receive more attention, but the study on subsequent impact of the extended technology model, especially the impact of the FACS on school identity, is scarce. In addition, students’ sense of identity (e.g., social identity, scientific identity, ethnic identity) has been discovered by predecessors to predict subsequent sense of belonging, but there is still a lack of research in the school field. Especially in the context of the COVID-19, there is a lack of research on TAM, school identity and school belonging from the integrated perspective.

**FIGURE 1:**
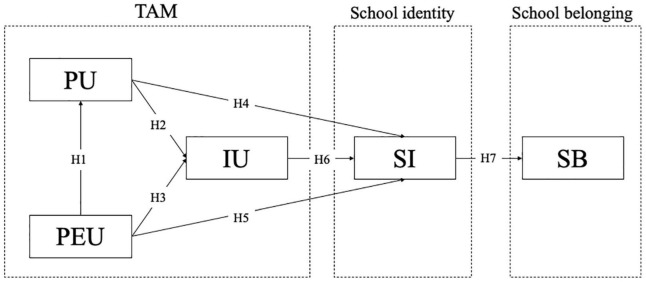
Hypothesis model of the relationship between the use of FACS, school identity, and school belonging. PU, Perceived usefulness; PEU, Perceived ease of use; IU, Intention to use; SI, School identity; SB, School belonging.

Due to COVID-19, many colleges and universities in China have installed FACS. To understand its influence on students’ school identity and school belonging, and to provide practical interventions and policy enlightenment for school, this research will use the structural equation modeling method to explore the relationship between college students’ technical acceptance, school identity and school belonging when using FACS. To this end, based on TAM, halo effect and social information processing theory, we investigated the use of FACS by college students using psychometric scales. By answering two questions: (a) How does TAM affect school identity and (b) How does school identity predict school belonging, we try to reveal the relationship between college students’ technical acceptance of FACS, school identity and school belonging, and explore the interactions and mechanisms. The hypothetical model of the relationship between access control system use, school identity and school belonging are shown in Figure 1. All the hypotheses 1∼7 are summarized as follows:

H1: PEU positively affects PU;

H2: PU positively affects IU;

H3: PEU positively affects IU;

H4: PU positively affects school identity;

H5: PEU positively affects school identity;

H6: IU positively affects school identity;

H7: School identity positively predicts school belonging.

## Materials and Methods

### Participants

In this study, a convenience sampling method was adopted to collect data from college students in China, from November 1 to November 15, 2021 using the Questionnaire Star online questionnaire platform. All the participants volunteered to participate and were told that the study was for academic purposes and that participants would receive no compensation of any kind. A sample of 400 college students in China was recruited to participate in the data collection. All participants were informed that the survey was voluntary and anonymous before filling out the questionnaire. After exclusions due to missing data and repeated answers, the effective sample size was 391. The average age of the sample was 22.41 years (*SD* ± 2.38). The sample included 297 girls (76%) and 94 boys (94%).

### Measures

The items of the questionnaire were adapted from previous theories or researchers. The original items were professionally translated into Chinese using the forward-backward method to verify the accuracy of the questionnaire and ensure the surface validity of the questionnaire.

#### Questionnaire of Perceived Usefulness

The PU questionnaire (Davis et al., 1989) was used to assess students’ PU toward FACS. The survey included four items (e.g., The use of FACS can reduce my time to access the school entrance). Students were requested to indicate the extent of PU on a scale from 1 (*strongly disagree*) to 5 (*strongly agree*) with higher scores indicating higher PU of FACS. The fitting indices indicated the structural validity of the model was acceptable (χ^2^/*df* = 0.953/1, CFI = 1.000, TLI = 1.000, RMSEA = 0.000, SRMR = 0.006). The reliability was accepted (Cronbach’s α = 0.97) from previous studies (Davis, 1989), and the Cronbach’s α in the current study was 0.81.

#### Questionnaire of Perceived Ease of Use

The PEU questionnaire (Davis et al., 1989) was used to examine the extent of students’ perceived ease to use the FACS. In total, there were four items (e.g., The interaction between FACS and myself is clear and easy to understand). Participants rated the items on a five-point Likert scale (1 = *strongly disagree*, 5 = *strongly agree*), with a higher score corresponding to a higher PEU. The internal consistency of this scale showed satisfactory reliability both in previous study (Cronbach’s α = 0.91) (Davis, 1989) and current study (Cronbach’s α = 0.91), and the structural validity of model was good to acceptable (χ^2^/*df* = 6.842/1, CFI = 0.994, TLI = 0.967, RMSEA = 0.122, SRMR = 0.009).

#### Questionnaire of Intention to Use

Two items were used to describe IU toward access control systems on a 5-point Likert scale. Students were asked to indicate the tendency of their use of the FACS, such as “the FACS worries me a lot.” Participants rated the items on a 5-point Likert scale (1 = *strongly disagree*, 5 = *strongly agree*), with a higher score indicating a higher tendency toward using FACS. The internal consistency of this scale showed satisfactory reliability (Cronbach’s α = 0.90) in current study and prior study (Cronbach’s α = 0.84) (Davis et al., 1989).

#### Questionnaire of School Identity

Students reported on their identity of school with a four-dimension questionnaire (i.e., group cognition, emotional dimension, evaluation dimension, and autonomous behavior) adapted from the scale of school identity (Ding, 2012). It includes 20-item using a five-point Likert scale (1 = *strongly disagree*, 5 = *strongly agree*), with a higher score indicating a higher sense of school identity. Sample item was “I think the image of my school reflects my image to some extent.” The scale demonstrated satisfactory internal consistency in current study (Cronbach’s α = 0.97) and previous study (Cronbach’s α = 0.875) (Ding, 2012), and the structural validity of model was good to acceptable (χ^2^/*df* = 7.666/2, CFI = 0.996, TLI = 0.988, 0.085, SRMR = 0.009).

#### Questionnaire of School Belonging

An adapted measure was used to assess the school belonging. This measure consisted of four dimensions (i.e., sense of belonging with peers, instructor support, engagement in the community, and relatedness of self with school) with a total of 22 items from the questionnaire (Mueller, 2008). And it uses a five-point Likert scale (1 = *strongly disagree* to 5 = *strongly agree*) with higher score indicating a higher sense of school belonging. Sample item is “I feel comfortable sharing thoughts, opinions, or feelings with other students at this university.” This measure had adequate internal consistency (Cronbach’s α = 0.94) in current study and previous study (Cronbach’s α = 0.75–0.89) (Mueller, 2008), and the structural validity of model was acceptable (χ^2^/*df* = 1.202/1, CFI = 1.000, TLI = 0.999, RMSEA = 0.023, SRMR = 0.004).

### Data Analyses

Firstly, Means, standard deviations, Kendall correlations, and Pearson correlations were calculated by using SPSS 20.0. Secondly, the hypothesized multiple mediation model was tested by structural equation modeling (SEM) using Mplus 7.4. SEM models enable scholars to evaluate complex models with regard to compatibility with all the relationships in the data set. And SEM based modeling enables more precise evaluation of indicator variable loadings as well as reliability and validity of measurement models (Astrachan et al., 2014). The model was evaluated by following model fitting indices: the chi-square values (χ^2^), the comparative fit index (CFI), the Tucker–Lewis fit index (TLI), the root mean square error of approximation (RMSEA), and the standardized root mean square residual (SRMR). The CFI and TLI at 0.90 or above, and the RMSEA and SRMR at 0.08 or lower, indicating that the model was acceptable (Hu and Bentler, 1999).

## Results

### Descriptive Statistics and Correlations

Means, standard deviations, and Pearson correlations were presented in Table 1. As shown, PU was significantly and positively correlated with PEU, IU, school identity, and school belonging. Furthermore, each two elements of PEU, IU, school identity, and school belonging had a positive association.

**TABLE 1:** Means, standard deviations, and correlations among the main variables.

Variables	*M*	*SD*	PU	PEU	IU	SI	SB	Gender	Age
PU	3.45	0.81	-						
PEU	3.47	0.79	0.77***	-					
IU	3.44	0.79	0.64***	0.73***	-				
SI	3.52	0.68	0.55***	0.56***	0.55***	-			
SB	3.50	0.52	0.49***	0.53***	0.48***	0.87***			
Gender	-	-	0.05	0.07	0.07	0.07	0.05		
Age	22.41	2.38	0.00	0.02	0.04	0.05	0.07	-0.22**	

PU, Perceived usefulness; PEU, Perceived ease of use; IU, Intention to use; SI, School identity; SB, School belonging. **p < 0.01, ***p < 0.001.

### Examinations of the Measurement Model

Before testing the hypothesized model by SEM, it was necessary to examine the measurement model. According to the recommendation from Wu and Wen (2011), PU, PEU and IU were averaged and treated as manifest variables, respectively. Both school identity and school belonging could both be loaded by their four observed substructures. Altogether, the CFA results of the measurement model showed a good model fit: χ^2^/*df* = 409.010/122, CFI = 0.954, TLI = 0.942, RMSEA = 0.078, SRMR = 0.046, in that all the loadings on latent variables were significant (*p* < 0.001).

### Examinations of the Structural Model

**FIGURE 2:**
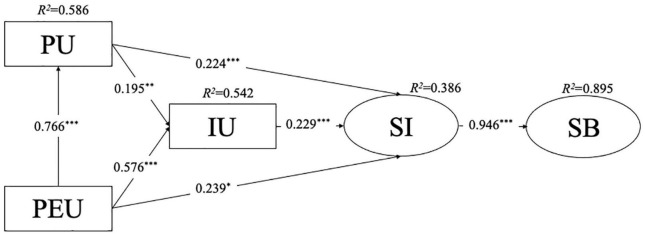
Research model results. *p < 0.05, **p < 0.01, ***p < 0.001.

As hypothesized, a hypothesized model was established with PU and PEU as the endogenous variable, IU and school identity as the mediators, as well as school belonging as the exogenous variable. The SEM results showed a good model fit: χ^2^/*df* = 121.654/36, CFI = 0.979, TLI = 0.967, RMSEA = 0.078, SRMR = 0.031. As shown in Figure 2, PU and PEU significantly predicted IU and school identity. Furthermore, PU and PEU positively predicted school identity through IU playing mediators. Finally, school identity did directly predict school belonging.

### Path Analysis

To further examine the significance of the indirect effects, bias-corrected bootstrap tests derived with 2,000 samples were used. The fact that the 95% confidence interval did not contain zero indicated statistical significance. As shown in Tables 2, 3, school identity significantly mediated the association between PEU and school belonging, supporting H1. Similarly, PU and school identity significantly mediated the association between PEU and school belonging, supporting H2. What is more, IU and school identity did also mediate the association between PEU and school belonging, supporting H3. Finally, PU, IU and school identity significantly mediated the association between PEU and school belonging, supporting H4.

**TABLE 2:** Results of H1∼H7.

Hypothesis	Results
H1: PEU positively affects PU	Supporting
H2: PU positively affects IU	Supporting
H3: PEU positively affects IU	Supporting
H4: PU positively affects school identity	Supporting
H5: PEU positively affects school identity	Supporting
H6: IU positively affects school identity	Supporting
H7: School identity positively predicts school belonging	Supporting

**TABLE 3:** Bias-corrected bootstrap tests on the direct and indirect effects.

Paths	Standardized (β)	95% Confidence interval		Hypothesis
		Low	High	
PEU→SI→SB	0.129	0.029	0.223	Supporting
PEU→PU→SI→SB	0.093	0.036	0.153	Supporting
PEU→IU→SI→SB	0.071	0.027	0.123	Supporting
PEU→PU→IU→SI→SB	0.018	0.005	0.037	Supporting

## Discussion

In the context of COVID-19, many new changes have taken place in school teaching methods and environmental facilities. To clearly explain the impact of this school service technology on students in the context of the epidemic, this research is the first to innovatively apply structural equation modeling, combined with the halo effect, social identity theory and social information processing theory to explore the acceptance of FACS of college students from an integrated perspective, and to clarify the relationship between acceptance, school identity and school belonging. Our research hypotheses have been confirmed by the research results, which are described below.

### Technology Acceptance Model Is Positively Associated With School Identity

In examining H1, H2 and H3, the results showed that the college students’ PEU of the FACS positively predicts PU (H1); PU positively predicts IU (H2); and PEU positively predicts the IU (H3). This finding is consistent with previous research on TAM (Davis, 1989; Davis et al., 1989; Lederer et al., 2000). In addition, according to the average scores of PEU, PU, and IU (PU = 3.45; PEU = 3.47; IU = 3.44), college students who rated highly of PEU and PU of FACS show strong willingness to use.

Since the original intention of the technology is to serve the needs of people, we further expanded the external influence of TAM on students, that is, exploring the influence of FACS on college students’ school identity. The results show that H4, H5 and H6 are all confirmed, that is, PEU, PU, and IU can positively and significantly predict the degree of school identity. Specifically, the higher the PEU, PU, and IU, the stronger their identity of their alma mater. This finding further validates and expands the application boundary of the halo effect in the field of school access control systems. Specifically, students’ perception of the school’s access control system will diffusely affect their overall school identity, which is in good agreement with previous studies (Thorndike, 1920; Ullman, 2015).

### School Identity Is Positively Associated With the Sense of Belonging

In examining H7, the results also showed that school identity enhanced by TAM further positively predicted school belonging, which means when students have stronger school identity during the epidemic, their school belonging is also stronger. This finding is consistent with the view of social identity theory (Schwartz, 2001). school identity is the concrete manifestation of identity in the school context. The higher the school identity, the more likely a student will see himself as a member of the school community. In this study, school belonging is a combination of four senses-Belonging with Peers, Instructor Support, Engagement in the Community, and Relatedness of Self with School-which all showing consistent predictive power. That school identity predicts school belonging is in line with previous research (Legette and Kurtz-Costes, 2021).

Combined with the previous influence of TAM on school identity, the emotional safety theory believes that the normal operation of the individual’s affiliate system depends on the sense of security obtained from the outside (Davies et al., 2018). Combined with the research results, this study suggests that the safe access control system during the epidemic may be an important initial source of individual security. The safety function of this external device further enhances the individual’s school belonging to the group by enhancing the individual’s school identity.

## Conclusion

In the context of COVID-19, many new changes have taken place in school teaching methods (Yang et al., 2020) and environmental facilities. To explore the impact of the use of the access control system on college students’ perception of campus, Structural Equation Modeling was conducted to provide quantitative research evidence. Empirically, the use of the access control system has an impact on college students’ school belonging and school identity. These findings are supported by theories of TAM, halo effect, and social identity from an integrated perspective. Also, this research has profound significance and has made good contributions to enriching current theory and practice. Specifically, it expands the application scenarios and follow-up conditions of TAM, and found that local conditions of the school will affect the overall perception of school. More importantly, the FACS not only protects the safety of teachers and students during the COVID-19 pandemic, but also strengthens students’ school identity and school belonging.

### Contributions

Theoretically, this study (a) expands the application scenarios and follow-up conditions of TAM. Specifically, the acceptance model of FACS was established, and the influence of this model on school identity was investigated as well. Among them, PU, PEU, and IU have all been proved to be the antecedents of school identity (Ullman, 2015). What’s more, this theory extends the theoretical boundary of the halo effect to the school identity, that is, the local conditions of the school will affect the overall perception of school; (b) this research has enriched the theoretical connotation and influence of school identity. It is found that school identity is an important source of school belonging, and this relationship is extremely stable (Legette and Kurtz-Costes, 2021).

Practically, this study provides strong evidence to support the promotion of school access control systems during the pandemic. The FACS not only protects the safety of teachers and students, but also enhances students’ school identity and school belonging during the outbreak. On the basis of previous research (Sanders and Munford, 2016; Verhoeven et al., 2019), this research provides a reference on how to cultivate college students’ school identity and school belonging in the aspect of school environmental facilities construction. For example, schools should improve the ease of use and usefulness of service facilities, improve the fluency of human-computer interaction, and make people be more willing to use them.

### Limitations and Future Study

Although this research has made some new contributions, there are still some shortcomings. Firstly, this study adopts a cross-sectional research design, and it is impossible to obtain an accurate causal mechanism due to the failure to manipulate variables. Secondly, the research objects of this study are limited to college students, and there is no survey on the school identity and school belonging of students in other school levels (such as primary and secondary schools, kindergartens), so the generalization of the conclusions may be limited. Lastly, the application of FACS also brings some hidden worries to the public, such as information leakage anxiety. The face recognition technology in this study is limited to the application of access control systems, so there may be insufficient explanations in other fields. In order to make up for the above shortcomings, future research can consider the use of longitudinal tracking design or laboratory research methods to investigate the causal mechanism between variables. In addition, the school identity and school belonging of primary, secondary, and kindergarten students are also particularly important, and we need to pay more attention to diverse student groups in the future. Finally, we hereby appeal that researchers should pay more attention to the impact of face recognition technology in other fields.

## Data Availability Statement

The original contributions presented in the study are included in the article/supplementary material, further inquiries can be directed to the corresponding author/s.

## Ethics Statement

Ethical review and approval was not required for the study on human participants in accordance with the local legislation and institutional requirements. Written informed consent for participation was not required for this study in accordance with the national legislation and the institutional requirements.

## Author Contributions

QW and XY: conceptualization. QW, LH, and J-CH: methodology. LH and QW: formal analysis and investigation. QW: writing—original draft preparation. XY: writing—review and editing and resources. MZ: supervision. All authors contributed to the article and approved the submitted version.

## Conflict of Interest

The authors declare that the research was conducted in the absence of any commercial or financial relationships that could be construed as a potential conflict of interest.

## Publisher’s Note

All claims expressed in this article are solely those of the authors and do not necessarily represent those of their affiliated organizations, or those of the publisher, the editors and the reviewers. Any product that may be evaluated in this article, or claim that may be made by its manufacturer, is not guaranteed or endorsed by the publisher.
